# Effect of Low-Frequency Repetitive Transcranial Magnetic Stimulation on Impulse Inhibition in Abstinent Patients With Methamphetamine Addiction: A Randomized Clinical Trial

**DOI:** 10.1001/jamanetworkopen.2020.0910

**Published:** 2020-03-13

**Authors:** Jiajin Yuan, Weijun Liu, Qiongdan Liang, Xinyu Cao, Molly V. Lucas, Ti-Fei Yuan

**Affiliations:** 1 Institute of Brain and Psychological Sciences, Sichuan Normal University, Chengdu, China; 2 Key Laboratory of Cognition and Personality of Ministry of Education, Faculty of Psychology, Southwest University, Chongqing, China; 3 Da Lian Shan Institute of Addiction Rehabilitation, Nanjing, China; 4 Wu Tsai Neurosciences Institute, Stanford University, Stanford, California; 5 Shanghai Key Laboratory of Psychotic Disorders, Shanghai Mental Health Center, Shanghai Jiao Tong University School of Medicine, Shanghai, China; 6 Co-innovation Center of Neuroregeneration, Nantong University, Nantong, Jiangsu, China

## Abstract

**Importance:**

Impulsivity during periods of abstinence is a critical symptom of patients who use methamphetamine (MA).

**Objective:**

To evaluate changes in impulse inhibition elicited by repetitive transcranial magnetic stimulation (rTMS) in patients with MA addiction.

**Design, Setting, and Participants:**

This randomized clinical trial was conducted in Da Lian Shan Addiction Rehabilitation Center, Nanjing, China, from December 1, 2018, to April 20, 2019. Effects of the intervention were examined at 3 time points: after a single session (day 1), 24 hours after 10 repeated sessions (day 11), and at 3 weeks of follow-up (day 31). Men with MA addiction and healthy male control participants were recruited for this study. Data analysis was performed from March 2019 to October 2019.

**Interventions:**

Patients who use MA were randomized to undergo sham rTMS (36 patients) and or 1-Hz rTMS (37 patients) to the left prefrontal cortex, receiving daily TMS treatments for 10 consecutive days.

**Main Outcomes and Measures:**

The primary outcome was impulse inhibition, which is primarily embodied by accuracy reduction (ie, accuracy cost) from standard to deviant trials in a 2-choice oddball task (80% standard and 20% deviant trials).

**Result:**

The study included 73 men with MA addiction (mean [SD] age, 38.49 [7.69] years) and 33 male healthy control participants without MA addiction (mean [SD] age, 35.15 [9.68] years). The mean (SD) duration of abstinence for the men with MA addiction was 9.27 (4.61) months. Compared with the control group, patients with MA addiction exhibited greater impulsivity (accuracy cost, 3.3% vs 6.2%). The single session of 1-Hz rTMS over the left prefrontal cortex significantly increased accuracy from 91.4% to 95.7% (*F*_1,36_ = 9.58; *P* < .001) and reaction time delay from 50 milliseconds to 77 milliseconds (*F*_1,36_ = 22.66; *P* < .001) in deviant trials. These effects were seen consistently after 10 sessions of 1-Hz rTMS treatment (day 11 vs day 1, *t*_26_ = 1.59; *P* = .12), and the behavioral improvement was maintained at least for 3 weeks after treatment (day 31 vs day 1, *t*_26_ = 0.26; *P* = .80). These improvement effects of impulse inhibition were coupled with a reduction in addictive symptoms as measured by cue-induced craving. The pretest accuracy cost was positively correlated with the change in impulse inhibition (*r* = 0.615; *P* < .001) and change in craving (*r* = 0.334; *P* = .01), suggesting that these 2 behaviors may be modified simultaneously.

**Conclusions and Relevance:**

These findings suggest that repeated rTMS sessions have sustained effects on impulse inhibition in patients with MA addiction and provide novel data on impulsivity management strategies for addiction rehabilitation.

**Trial Registration:**

ChiCTR-ROC-16008541

## Key Points

**Question:**

Is low-frequency repetitive transcranial magnetic stimulation associated with reduced behavioral impulsivity in patients who use methamphetamine?

**Findings:**

In this randomized clinical trial, patients who use methamphetamine exhibited greater impulsivity than healthy control participants, and low-frequency repetitive transcranial magnetic stimulation over the left prefrontal cortex improved impulsivity control in these patients.

**Meaning:**

These findings suggest that brain stimulation could be adopted for impulsivity intervention in the rehabilitation of patients who use methamphetamine.

## Teaser

This randomized clinical trial evaluates changes in impulse inhibition elicited by repetitive transcranial magnetic stimulation in patients with methamphetamine addiction.

## Introduction

Drug addiction is characterized by compulsive drug-seeking behavior.^1^ The level of impulsivity in an individual is a prominent factor in both the propensity to use drugs and the susceptibility to becoming addicted in later stages of addiction formation.^2^ Impulsivity or poor behavioral inhibition could be associated with many addictive behaviors and may be particularly important at certain phases of addiction formation.^3^ Methamphetamine (MA) dependence is associated with increased impulsivity,^4^ which is attributed to several neural circuit changes, such as aberrant midbrain-ventral striatum resting connectivity and reduced striatal dopamine receptor availability.^5,6^ Regaining behavioral control and decreasing impulsivity are critical steps for successful rehabilitation from MA dependence.^7^

The prefrontal cortex (PFC) plays a pivotal role in the execution and inhibition of behavioral actions. It is implicated in decision-making processes, such as the inhibition of risk-taking options. Dysfunction of the PFC has been implicated in psychostimulant dependence and is thought to cause a loss of behavioral control.^8^ For instance, patients with MA addiction show poor performance in working memory, cognitive control, attention, and decision-making processes, all of which are closely associated with neuroimaging measurements of the PFC system (eg, regional metabolism, blood flow, and activation).^9^ Notably, recent evidence shows that reactivation of the PFC with noninvasive brain stimulation (eg, repetitive transcranial magnetic stimulation [rTMS]) could reduce cue-induced craving and drug intake in different types of drug addiction, including MA, cocaine, heroin and nicotine.^10,11,12,13^ However, whether rTMS treatment could reduce behavioral impulsivity remains to be elucidated.

A previous study^14^ showed that high-frequency (20 Hz) rTMS over the left dorsolateral PFC increases activation in subcortical emotional circuits (eg, amygdala, insula, and basal ganglia), which mediate hyperarousal and behavioral impulsivity.^15,16^ In contrast, low-frequency (1 Hz) stimulation of the dorsolateral PFC increases activation in the anterior cingulate cortex and bilateral parietal cortices,^17^ 2 key nodes of the frontoparietal cognitive control network.^18,19^ In addition, low-frequency rTMS at the left PFC (eg, dorsolateral area) has proven to be effective in reducing the symptoms of attention-deficit/hyperactivity disorder and autism spectrum disorder, both of which include impulsive behavior patterns.^20,21^ These findings suggest that low-frequency rTMS at the left PFC may mitigate the impulsivity seen in patients with MA addiction.

This study aims to understand whether low-frequency rTMS stimulation over the left PFC can reduce behavioral impulsivity in patients with MA addiction, using a sham-controlled, double-blind intervention design. The 2-choice oddball task was used to quantify the behavioral impulsivity by providing multiple lines of behavioral data, as described previously.^22,23^ This task requires participants to respond to both frequent standard and infrequent deviant trials. The frequent presentation of standard trials leads participants to form a habitual response to the standard stimulus. As a result, participants must inhibit their habitual, prepotent response to respond accurately during deviant trials. This produces 2 outcomes: one is reduced accuracy for deviant vs standard conditions, whereas the other is delayed response in deviant vs standard trials when the response is accurate. Consequently, reaction time (RT) can be used to understand how accuracy is changed, which is absent in other tasks relying solely on accuracy to track impulse inhibition (eg, go or no-go task).

## Methods

### Participants

**Figure 1.**
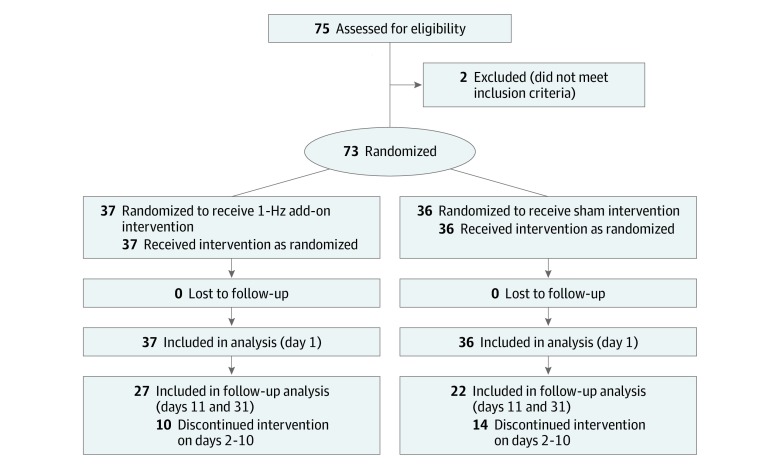
Study Flowchart

Seventy-three men from Da Lian Shan Addiction Rehabilitation Center who met the *Diagnostic and Statistical Manual of Mental Disorders* (Fifth Edition) criteria for MA dependence were recruited for the present study, according to a priori computation of the required sample size with the current design using G*Power statistical software version 3.1.9.4 (University of Dusseldorf), where 36 participants are necessary for 0.95 statistical power, and 73 participants were used in case of potential sample missing. The eligibility criteria included no mixed use of other drugs in addition to MA, no physical disability, no acute physical or psychiatric illness, willingness to cease MA use, and no hallucination and acute withdrawal symptoms. The exclusion criteria included mixed use of multiple drugs, current medical conditions and medicine use, hallucination or acute withdrawal symptoms in the past 4 weeks, and obvious physical disability. Each participant was randomly assigned to either the 1-Hz rTMS treatment (37 participants) or sham rTMS treatment (36 participants), according to a computer-generated randomization sequence (Figure 1).

Thirty-three healthy male participants were recruited as the healthy controls (HCs). Demographic variables were matched with the MA group in terms of age, education, alcohol use, and smoking habits (Table).

**Table.** Demographic Data of Patients With Methamphetamine Addiction Undergoing 1-Hz or Sham rTMS and Healthy Controls

Characteristic	Patients With Methamphetamine Addiction		Healthy Controls	*F*/χ^2^	*P* Value
	1-Hz rTMS	Sham rTMS			
Sex	Male	Male	Male	NA	NA
Participants, No.	37	36	33	NA	NA
Age, mean (SD), y	39.37 (8.08)	37.58 (7.26)	35.15 (9.68)	2.23	.11
Education^a^	3.0 (1.5)	2.5 (1.0)	3.0 (1.5)	2.84	.06
Smoking, %^b^	97.29	86.11	84.84	3.63	.16
Alcohol use, %^b^	59.46	61.11	66.67	0.42	.81

Abbreviations: NA, not applicable; rTMS, repetitive transcranial magnetic stimulation.

^a^ Education is shown as median (interquartile range [maximum minus minimum]) level, denoted as 1 for primary school, 2 for junior high school, 3 for senior high school, 4 for college, and 5 for postgraduate.

^b^ Refers to use before rehabilitation.

All the participants participated in the study voluntarily and provided written informed consent. The study was approved by local ethics committee of human research at Southwest University and Sichuan Normal University in China. The experimental procedure was in accordance with the ethical principles of the 1964 Declaration of Helsinki. This study follows the Consolidated Standards of Reporting Trials (CONSORT) reporting guidelines. The trial protocol is shown in Supplement 1.

All participants were naive to TMS treatment. Behavioral procedures and rTMS interventions were administered by researchers who were blind to the research purposes. Outcome measures were assessed by independent researchers blinded to treatment group assignment.

### rTMS Procedure

For real or sham rTMS, the motor threshold was determined by stimulating the left primary motor cortex. The TMS coil was placed over the left primary motor cortex in posterior-anterior direction, and the lowest intensity that elicits a motor response in the right abductor pollicis brevis muscles (≥5 muscle contractions in 10 trials) was taken as the resting motor threshold.^24,25^ During the rTMS procedure, the coil was placed over the stimulation site of the left PFC, which was defined as being 5 cm anterior to the thumb region for left primary motor cortex, corresponding to the left dorsolateral PFC, as described elsewhere.^24,26^ Low-frequency (1 Hz; 100% resting motor threshold intensity; 600 pulses over 10 minutes) or sham (coil turned away from the skull at 90°, resting on the scalp with 1 edge) rTMS was applied over the left PFC according to group assignment. The validity of this sham protocol has been verified by prior studies.^10,25^ We used a figure 8–shaped coil (radius of 45 mm for each circle; the center distance between 2 circles is 76 mm) for accurately targeted stimulation using a magnetic-electric stimulator (CCY-I TMS; Yiruide Co). The rTMS session was performed for 10 consecutive days. The coil position was maintained within and across sessions, and a patient-specific TMS cap was used for TMS navigation.

### Behavioral Measurement

For the 2-choice oddball task, each trial started with a jittered fixation cross, varying from 500 milliseconds to 1500 milliseconds. Following this, the task stimulus was presented. For one-half of the participants in each treatment group, if the standard stimulus (“W”; 80% of trials) was presented, they were to press “F” with their left index finger as quickly as possible. If the task stimulus was the deviant stimulus (“M”; 20% trials), they were to press the “J” key with their right index finger. For the second half of the participants, the response keys were reversed (ie, they were to press “J” for standard stimuli and “F” for deviant stimuli). Before the task starting, each participant completed 15 practice trials to familiarize them with the procedure. To avoid the practice effect, the formal experiment did not start until participants achieved 100% accuracy for both standard and deviant stimuli during practice. At the end of the experiment, participants were told their accuracy as feedback about their performance. Behavioral impulsivity was primarily indicated by the levels of accuracy reduction during deviant vs standard trials. The RT delay during deviant vs standard trials was also recorded to provide context for how accuracy is altered (accuracy improvement was positively associated with RT delay) (eFigure 1 in Supplement 2).^27^

**Figure 2.**
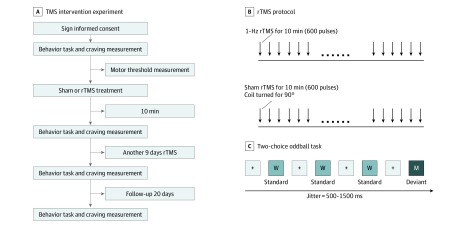
Study Design A, Flowchart shows study design of transcranial magnetic stimulation (TMS) intervention. B, Illustration of 1-Hz real or sham repetitive TMS (rTMS) protocols. C, Illustration of behavioral procedure of 2-choice oddball task with a standard stimulus (W) and a deviant stimulus (M).

A cue-induced craving test was performed after the 2-choice oddball task, by asking patients with MA addiction to attend to an MA-intake video for 5 minutes. Participants were instructed to watch the video carefully and then rated their level of craving for MA intake, using a visual analog scale ranging from 0 (not at all) to 100 (extremely intense). The behavioral procedure lasted for approximately 12 minutes, including the 2-choice oddball task and cue-induced craving test. The behavioral procedure was administered 5 minutes before and after the first rTMS session (day 1) and 24 hours after the last rTMS session (day 11). A 3-week follow-up test was administered on day 31 (Figure 2).

### Statistical Analysis

To analyze the impulse inhibition of patients with MA addiction compared with that of HCs, a mixed-design analysis of variance model was used with stimulus (2 levels, standard and deviant) as a repeated factor and group (HC and MA) as a between-participant variable. To assess the effects of rTMS on impulse inhibition in patients with MA addiction, a mixed-design analysis of variance was used with stimulus (2 levels, standard and deviant) and time (2 levels, before and after) as repeated variables with TMS (real 1-Hz rTMS and sham rTMS) as a between-participants variable. The degrees of freedom of the *F* ratio were corrected for violation of spherical assumption according to the Greenhouse-Geisser method. The Bonferroni-Holm method was used for post hoc comparisons if statistically significant main or interaction effects appeared, or if sustained intervention effects that involve day 1 and day 11 (or day 31) comparisons were examined. The data analysis was conducted using SPSS statistical software version 20.0 (IBM). A 2-sided *P* < .05 was considered statistically significant, and the effect size was reported as partial η^2^ (η^2^_p_). Data analysis was performed from March 2019 to October 2019.

## Results

### Participant Characteristics

The mean (SD) age of the 33 HCs was 35.15 (9.68) years. The overall mean (SD) age of the 73 men with MA addiction was 38.49 (7.69) years (mean [SD], 39.37 [8.08] years for the 37 participants assigned to the 1-Hz rTMS treatment and 37.58 [7.26] years for the 36 participants assigned to the sham rTMS treatment). The mean (SD) abstinence duration was 9.27 (4.61) months, and the 2 groups were overall matched in the duration of abstinence (8.4 months for 1-Hz and 10.1 months for sham; difference, 1.7 months; 95% CI, −15.2 to 12.6 months; *P* = .11).

### Manipulation Check

**Figure 3.**
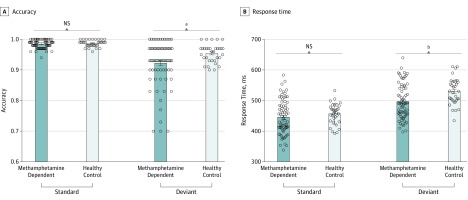
Comparison of Impulsivity-Inhibitory Performance Between Healthy Controls and Patients With Methamphetamine Addiction Before Repetitive Transcranial Magnetic Stimulation A and B, The 73 patients with methamphetamine addiction compared with 33 healthy controls showed more accuracy reduction (A) and smaller response time delay (B) during deviant compared with standard trials, indicating increased impulsivity. Bars denote means, error bars denote standard errors of the mean, and circles denote individual data points. NS indicates not statistically significant. ^a^*P* = .02. ^b^*P* = .004.

The analysis of accuracy and RT data in the 2-choice oddball task showed statistically significant group-by-stimulus interaction effects (accuracy, *F*_1,104_ = 5.01, *P* = .03, and η^2^_p_ = 0.05; RT, *F*_1,104_ = 13.00, *P* = .001, and η^2^_p_ = 0.11). Accuracy was reduced in deviant compared with standard trials for both samples (patients with MA, *t*_72_, −7.36; HCs, *t*_32_, −6.64; *P* < .001 for both), whereas this decrease was more pronounced in patients with MA addiction compared with HCs. The mean accuracy cost was 3.3% for HCs and 6.2% for patients with MA addiction. Although patients with MA addiction and HCs showed a similar accuracy ceiling for standard trials (98.4% vs 98.7%; *t*_104_, −1.13; *P* = .26), the accuracy for deviant trials was significantly lower in patients with MA addiction compared with HCs (92.2% vs 95.4%; *t*_104_, −2.39; *P* = .02) (Figure 3). The RT was longer for deviant compared with standard trials (patients with MA, *t*_72_, 14.17; HCs, *t*_32_, 15.55; *P* < .001 for both). The RT delay was less pronounced in patients with MA addiction (RT delay, 52 milliseconds) compared with HCs (RT delay, 73 milliseconds). Although patients with MA addiction and HCs showed similar RTs for standard trials (RT, 445 vs 456 milliseconds; *t*_104_, −1.04; *P* = .30), the RTs for deviant trials were significantly faster for patients with MA addiction compared with HCs (RT, 496 milliseconds vs 530 milliseconds; *t*_104_, −2.99; *P* = .004) (Figure 3). Compared with HCs, patients with MA addiction showed impaired impulse inhibition, with fast response at the sacrifice of the goal of maintaining accuracy (eFigure 1 in Supplement 2).

### Intervention Effects: 2-Choice Oddball task

The analysis of accuracy data showed a statistically significant 3-way interaction among stimulus, time, and group (*F*_1,71_ = 11.09; η^2^_p_ = 0.14; *P* = .001). The simple effect analysis shows a statistically significant stimulus-by-time interaction (*F*_1,36_ = 9.58; η^2^_p_ = 0.21; *P* = .004) in the 1-Hz real rTMS sample. Although behavioral accuracy was improved for both standard (99.4% vs 98.4%; *t*_36_, 4.10; *P* < .001) and deviant (95.7% vs 91.4%; *t*_36_, 4.01; *P* < .001) trials, this intervention-related improvement was more pronounced in deviant trials. However, the stimulus-by-time interaction was not statistically significant in the sham group (*F*_1,35_ = 3.89; *P* = .06).

**Figure 4.**
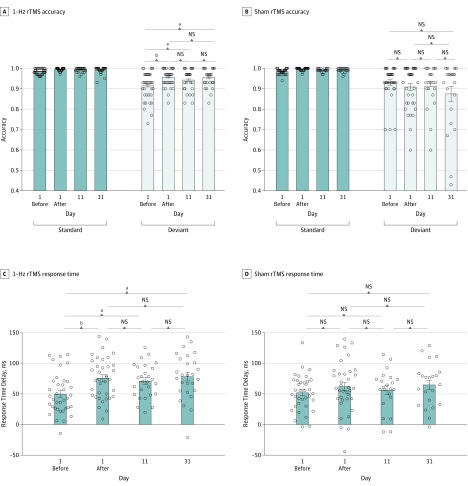
Accuracy and Reaction Time of Real and Sham 1-Hz Repetitive Transcranial Magnetic Stimulation (rTMS) A-D, Graphs show immediate (day 1) and long-term (day 11 or 31) effects of 1-Hz real (A and C) or sham (B and D) rTMS on impulsivity inhibition performance measured by accuracy (A and B) and deviant minus standard response time delay (C and D). Bars denote means, error bars denote standard errors of the mean, and circles denote individual data points. NS indicates not significant. ^a^*P* < .05. ^b^*P* < .001.

As a result, the 1-Hz real rTMS group had a posttest accuracy comparable with the HC level (*t*_68_, 0.28; *P* = .78) despite lower accuracy in the pretest (*t*_68_, −3.22; *P* = .002). The retests on day 11 (*t*_26_, 1.59; *P* = .12) and day 31 (*t*_26_, 0.26; *P* = .80) showed a continued similar accuracy as in the posttest of day 1 (Figure 4).

To further explain how impulse inhibition improved after 1-Hz real rTMS, we analyzed the RT data, with stimulus, time, and group as factors in an analysis of variance. Again, the results showed a statistically significant 3-way interaction among stimulus, group, and time (*F*_1,71_ = 4.08; η^2^_p_ = 0.054; *P* = .047), with the stimulus-by-time interaction statistically significant for the 1-Hz rTMS group (*F*_1,36_ = 22.66; η^2^_p_ = 0.39; *P* < .001) but not for the sham rTMS group (*F*_1,35_ = 3.06; *P* = .09). For the 1-Hz real rTMS group, RT showed significant delays for deviant compared with standard trials both before (*t*_36_, 9.27; *P* < .001) and after (*t*_36_, 13.91; *P* < .001) rTMS. However, the RT delay was significantly longer after rTMS compared with before rTMS (RT, 77 vs 50 milliseconds; difference, 27 milliseconds; 95% CI, 15-37 milliseconds; *P* < .001) . This suggests that patients with MA addiction responded in a more conservative, goal-oriented manner to optimize accuracy after 1-Hz rTMS.

The RT delays measured on day 11 and on day 31 were consistent with that in the posttest of day 1. All were prolonged compared with that in the pretest (Figure 4).

### Intervention Effects: Cue-Induced Craving

There was a statistically significant time-by-group interaction effect (η^2^_p_ = 0.10; *P* = .03), with self-reports of cue-induced craving significantly reduced after compared with before 1-Hz rTMS (η^2^_p_ = 0.49; *P* < .001) but not sham rTMS. The retest for the 1-Hz group on day 11 (η^2^_p_ = 0.51; *P* < .001) and day 31 (η^2^_p_ = 0.58; *P* < .001) exhibited a continued lower craving than that in pretest (eFigure 2 in Supplement 2).

### Correlation Analyses

To explore whether impulse inhibition is associated with addictive behavior, we computed the correlations between cue-induced craving and impulse inhibition in the pretest. Impulse inhibition was measured by the accuracy cost (standard accuracy minus deviant accuracy) or RT delay (deviant RT minus standard RT) in the 2-choice oddball task. The results showed that the accuracy cost was positively correlated with cue-induced craving (*r* = 0.301; *P* = .02) (eFigure 3 in Supplement 2), suggesting that impulsivity was tightly associated with addiction behavior.

To examine whether alteration of impulse inhibition is related to changes in addictive behavior after rTMS, we calculated the change in impulse inhibition (accuracy cost before minus accuracy cost after) and change in craving (before minus after). Pretest accuracy cost was positively correlated with the change in impulse inhibition (*r* = 0.615; *P* < .001) and change in craving (*r* = 0.334; *P* = .01), suggesting that these 2 behaviors may be modified simultaneously.

## Discussion

To our knowledge, this is the first study comparing rTMS treatment with sham treatment for behavioral impulsivity in patients with MA addiction. These findings provide evidence that low-frequency (1-Hz) rTMS is effective in reducing impulsive behavior in this addiction population. The rTMS treatment not only improves the accuracy in response but also adjusts the patients’ response strategy to be more goal oriented. Repeated rTMS sessions produced lasting effects of treatment for at least 3 weeks beyond the final day of treatment. These findings indicate that rTMS protocol might serve as an intervention for impulsivity in patients with MA addiction.

Recent studies^20,21,28^ indicate that low-frequency rTMS produces inhibitory effects on cortical excitability,^28^ hyperactivity,^20^ and impulsivity^21^ in both healthy and clinical populations. Several potential mechanisms may subserve the current findings. First, it was reported that low-frequency rTMS of the dorsolateral PFC increased focused attention and prefrontal inhibitory functions in patients with autistism^21,29^ by enhancing prefrontal-parietal coherent activity.^30^ If a similar mechanism exists for patients with MA addiction, this may repair the functional deficits of prefrontal system due to long-term MA use.^31^ Second, it has been reported that 1-Hz rTMS decreased the activation of subcortical emotional circuit,^14^ which may help to reduce arousal and impulsivity. Third, MA use has been found to increase releases of dopamine from the striatum^32^ and to reduce the density of serotonin transporter in global brain areas, which is implicated in aggressive behaviors even in currently abstinent MA users.^33^These neurotransmitter alterations, together with metabolic deficits of PFC,^34^ may result in functional abnormalities in cortical and subcortical circuits vital in emotional and behavioral control.^32,33^ Thus, patients with MA addiction may have prefrontal dysregulation of subcortical areas, which requires an intervention protocol that rebuilds PFC-subcortical connectivity. Consistent with this hypothesis, there is evidence that 1-Hz rTMS over the left dorsolateral PFC elicited coherent activation of prefrontal-subcortical neural circuits in individuals with depression.^35^ However, given the many differences between these psychiatric disorders and MA addiction, brain mechanisms supporting the rTMS-related impulsivity intervention in attention-deficit/hyperactivity disorder and autism spectrum disorder may not be generalized to patients with MA addiction. Future study needs to explore neural mechanisms underlying the intervention effects on MA-related impulsivity using neuroimaging techniques.

With evidence that high-frequency rTMS reduces cue-induced or spontaneous craving,^10,11,36,37^ studies have reported associations of low-frequency rTMS of the left PFC with craving score.^38,39^ It is notable that these 2 studies reported inconsistent results concerning the associations of low-frequency rTMS with cue-induced craving in patients with MA addiction. This might be explained by the use of the small sample size in both studies. The findings of the present study, which used a multisession rTMS protocol and a larger sample size, suggest that low-frequency rTMS can reduce behavioral impulsivity, which is associated with craving changes that result from the rTMS treatment. Further efforts are needed to identify the mechanism of the current rTMS effects and the phenotypes that preferentially modulate impulsivity or craving in response to drug-related cues.

### Limitations

This study has several limitations. Although HCs performed the behavioral task at baseline to compare with the patients with MA addiction, they did not undergo rTMS. This left us unable to investigate how TMS alters impulse inhibition in healthy persons. In addition, the present study used the 5-cm rule for TMS navigation, which does not take individual anatomy into account when determining the stimulation site of the left PFC. Despite abundant evidence linking this protocol to the stimulation site of the dorsolateral PFC,^40^ this method was shown to be inaccurate in locating the dorsolateral PFC in some studies (eg, inaccurately targeting the premotor area).^25,41^ This issue should be overcome in future studies using magnetic resonance imaging–guided neuronavigation, considering that different prefrontal areas serve different roles in inhibitory control.^42^ Furthermore, the current study did not collect functional neuroimaging data with rTMS intervention, leaving it unknown what neural plasticity mechanisms support the improved impulse inhibition across 1-Hz rTMS. This needs to be further investigated using a functional magnetic resonance imaging technique in future studies. In addition, the behavior results on impulse inhibition could be further verified with other established tasks (eg, stop-signal task, go or no-go task).

## Conclusions

This study found that 1-Hz rTMS elicited effective inhibition on behavioral impulsivity of patients with MA addiction, and repeated treatment sessions produced lasting effects in follow-up tests. The low-frequency rTMS procedure in future might be replaced by other accelerated brain stimulation protocols (eg, continuous theta-burst magnetic stimulation), which could greatly facilitate the treatment courses and increase the capacity of treatment.

## Notes

Supplement 1. **Trial Protocol**

Supplement 2. **eFigure 1.** Scatterplots for the Correlation Between ACC Cost and RT Delay in the Pretest and the Correlation Between Delta ACC Cost and Delta RT Delay **eFigure 2.** Schematic Illustration of Cue-Induced Craving as a Function of the 1-Hz rTMS Protocol on Days 1, 11, and 31 **eFigure 3.** Scatterplots of the Correlations Between the ACC Cost and Cue-Induced Craving in the Pretest, Between Pretest ACC Cost and Delta Impulse Inhibition, and Between Pretest ACC Cost and Delta Craving

Supplement 3. **Data Sharing Statement**
