# Error in Funding/Support Footnote

**DOI:** 10.1001/jamanetworkopen.2020.4467

**Published:** 2020-04-07

In the Original Investigation titled “Effect of Low-Frequency Repetitive Transcranial Magnetic Stimulation on Impulse Inhibition in Abstinent Patients With Methamphetamine Addiction: A Randomized Clinical Trial,”^1^ published March 13, 2020, in the first sentence of the Funding/Support footnote, the numbers for the grants from the National Natural Science Foundation of China to Dr J. Yuan were incorrect. The correct numbers are 31971018 and 31671164. This article has been corrected.^1^

## References

[1] Yuan J, Liu W, Liang Q, Cao X, Lucas MV, Yuan T-F Effect of low-frequency repetitive transcranial magnetic stimulation on impulse inhibition in abstinent patients with methamphetamine addiction: a randomized clinical trial. JAMA Netw Open. 2020;3(3):e200910. doi:10.1001/jamanetworkopen.2020.0910 32167568 PMC7070234

