# Supplementary material for: Effect of Low-Frequency Repetitive Transcranial Magnetic Stimulation on Impulse Inhibition in Abstinent Patients With Methamphetamine Addiction: A Randomized Clinical Trial
Source: JAMA Netw Open. 2020 Mar 13;3(3):e200910. doi: 10.1001/jamanetworkopen.2020.0910 (PMC7070234; doi:10.1001/jamanetworkopen.2020.0910)
Supplement: Supplement 1. — Trial Protocol [file jamanetwopen-3-e200910-s001.pdf]

Protocol Title: Low-frequency repetitive TMS at prefrontal cortex improves impulse inhibition in methamphetamine dependents

Protocol Number (Clinical Trial Registration Identifier):

ChiCTR-ROC-16008541 at http://www.chictr.org.cn

Ethics and dissemination: The study was approved by local ethic committee of human research at Southwest University and Sichuan Normal University in China. The experimental procedure was in accordance with the ethical principles of the 1964 Declaration of Helsinki (World Medical Organization, 1996).

Name of funding source or sponsor: National Natural Science Foundation of China (31371042, 31400906) to JY and (81822017, 31771215) to TY.

1. Purpose of Project and Scientific Justification

Background: Drug addiction is characterized with compulsive drug seeking behavior. Individual impulsivity acts as one prominent feature that predicts the propensity to use drug and the susceptibility of becoming addicted in later stage. Likewise, Methamphetamine dependence is associated with increased impulsivity. Targeting impulsivity and regained behavioral control are of importance in rehabilitation from methamphetamine dependence.

Purpose: This study aims to understand whether low frequency rTMS stimulation over the left PFC can reduce behavioral impulsivity in methamphetamine (MA) dependents.

2. Criteria for Patient Selection

Each of them had a history of regular (every week) and exclusive (no mixed use of other drugs) use of methamphetamine for more than 1 year. The eligibility criteria included no mixed use of other drugs in addiction to MA, no

physical disability; no acute physical or psychiatric illness, willingness to cease MA use; as well as no hallucination and acute withdrawal symptoms.

3. Study Design, Outcome Measures, and Procedures

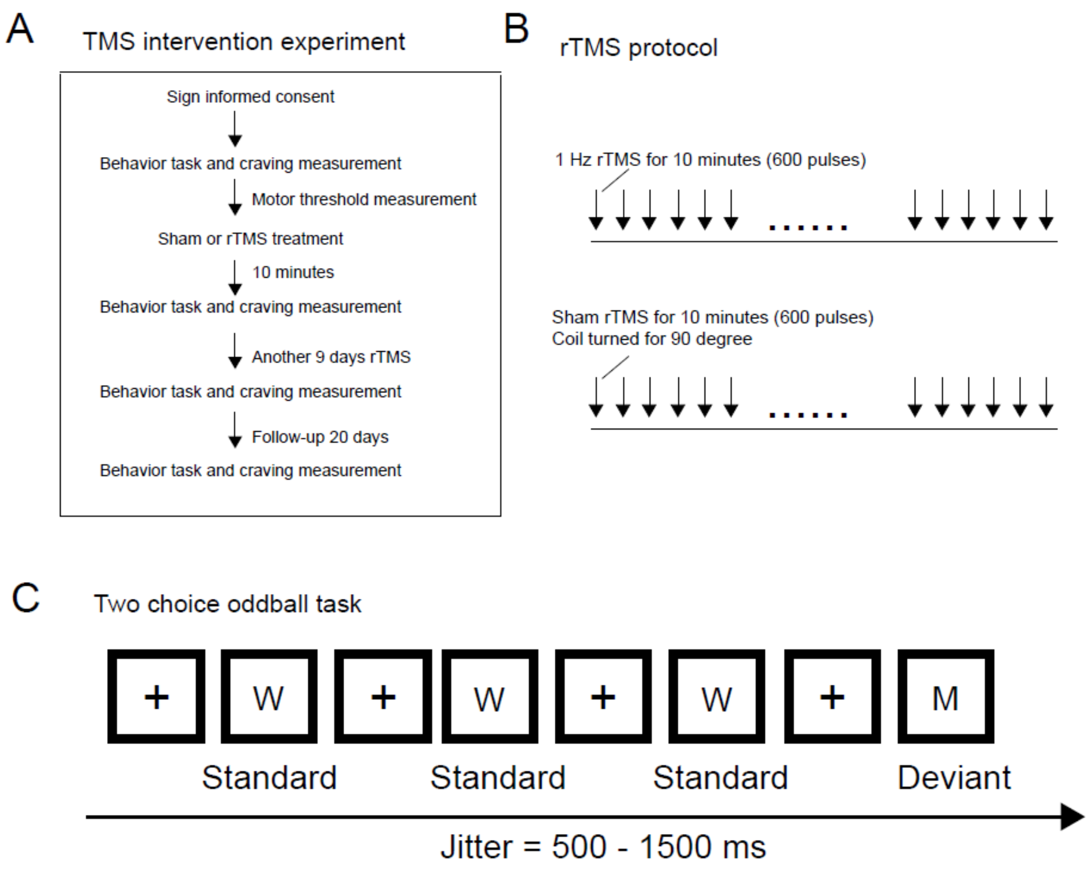

Main Measure： The Two-Choice Oddball Task stimulus was used to assess impulsivity control. For half of the subjects in each treatment group, if the task stimulus was the standard stimulus (“W”, 80% trials), they were to press “F” with their left index finger as quickly as possible. If the task stimuli was the deviant stimulus (“M”, 20% trials), they were to press the “J” key with their right index finger. For the second half of the participants, the response keys were reversed.

Secondary measure: A cue-induced craving test was performed after the two-choice oddball task, by asking MA dependents to attend to a methamphetamine-intake video for 5 minutes. Participants were instructed to watch the video carefully and then rated their level of craving for methamphetamine intake, using a visual analog scales (VAS) ranging 0 (not at all) and 100 (extremely intense).

The behavioral procedure was performed 5 minutes before the rTMS and repeated 5 minutes after the rTMS procedure for posttest day 1, as well as 24 hours after the last rTMS session (day 11). An unexpected behavioral test was administered on day 31.

Repetitive TMS Protocol: For rTMS procedure, Low frequency (1 Hz, 100% resting motor threshold intensity; 600 pulses over 10 minutes), or sham (coil turned away from the skull at 90 degrees, resting on the scalp with one edge) rTMS was applied over the left PFC according to group assignment. We used a figure-8-shaped coil (radius= 45mm for each circle, the center distance between two circles is 76mm) for accurately targeted stimulation using the CCY-I TMS instrument. The rTMS session was performed for 10 consecutive days. The coil position was maintained within and across sessions, and a patient-specific TMS cap was used for TMS navigation.

4. Statistical Analysis

To analyze MA patients' impulse inhibition compared to that of healthy controls (HC), a mixed-design ANOVA model was used with stimulus (2 levels: standard; deviant) as a repeated factor while Group as a between-subject variable (HC, MA).

To assess the effects of rTMS on impulse inhibition in MA patients, a mixed design ANOVA was used with Stimulus (2 levels: standard, deviant) and Time (2 levels: pre, post) as repeated variables while TMS (real rTMS (1Hz), sham rTMS) as a between-subjects variable. The degrees of freedom of the F-ratio were corrected for violation of spherical assumption according to the Greenhouse-Geisser method. Bonferroni-Holm method was used for post hoc comparisons. The data analysis was conducted using SPSS software (version 20.0). A 2-sided p-value less than 0.05 was considered statistically significant. Effect size was reported as partial eta square ($\eta 2_p$).

5. Consent Documentation

All the subjects participated in the study voluntarily and signed a written informed consent. The study was approved by local ethic committee of human research at Southwest University and Sichuan Normal University in China.

6. Alternatives to Participation

Subjects either do not receive any treatment in this study or forego treatment in order to participate in this study. The alternative, therefore, is not to participate.
