# Supplementary material for: Effect of Low-Frequency Repetitive Transcranial Magnetic Stimulation on Impulse Inhibition in Abstinent Patients With Methamphetamine Addiction: A Randomized Clinical Trial
Source: JAMA Netw Open. 2020 Mar 13;3(3):e200910. doi: 10.1001/jamanetworkopen.2020.0910 (PMC7070234; doi:10.1001/jamanetworkopen.2020.0910)
Supplement: Supplement 2. — eFigure 1. Scatterplots for the Correlation Between ACC Cost and RT Delay in the Pretest and the Correlation Between Delta ACC Cost and Delta RT Delay eFigure 2. Schematic Illustration of Cue-Induced Craving as a Function of the 1-Hz rTMS Protocol on Days 1, 11, and 31 eFigure 3. Scatterplots of the Correlations Between the ACC Cost and Cue-Induced Craving in the Pretest, Between Pretest ACC Cost and Delta Impulse Inhibition, and Between Pretest ACC Cost and Delta Craving [file jamanetwopen-3-e200910-s002.pdf]

Supplementary Online Content

Yuan J, Liu W, Liang Q, Cao X, Lucas MV, Yuan T-F. Effect of low-frequency repetitive transcranial magnetic stimulation on impulse inhibition in abstinent patients with methamphetamine addiction: a randomized clinical trial. *JAMA Netw Open*. 2020;3(3):e200910. doi: 10.1001/jamanetwork.open.2020.0910

eFigure 1. Scatterplots for the Correlation Between ACC Cost and RT Delay in the Pretest and the Correlation Between Delta ACC Cost and Delta RT Delay
eFigure 2. Schematic Illustration of Cue-Induced Craving as a Function of the 1-Hz rTMS Protocol on Days 1, 11, and 31
eFigure 3. Scatterplots of the Correlations Between the ACC Cost and Cue-Induced Craving in the Pretest, Between Pretest ACC Cost and Delta Impulse Inhibition, and Between Pretest ACC Cost and Delta Craving

This supplementary material has been provided by the authors to give readers additional information about their work.

© 2020 Yuan J et al. *JAMA Network Open*.

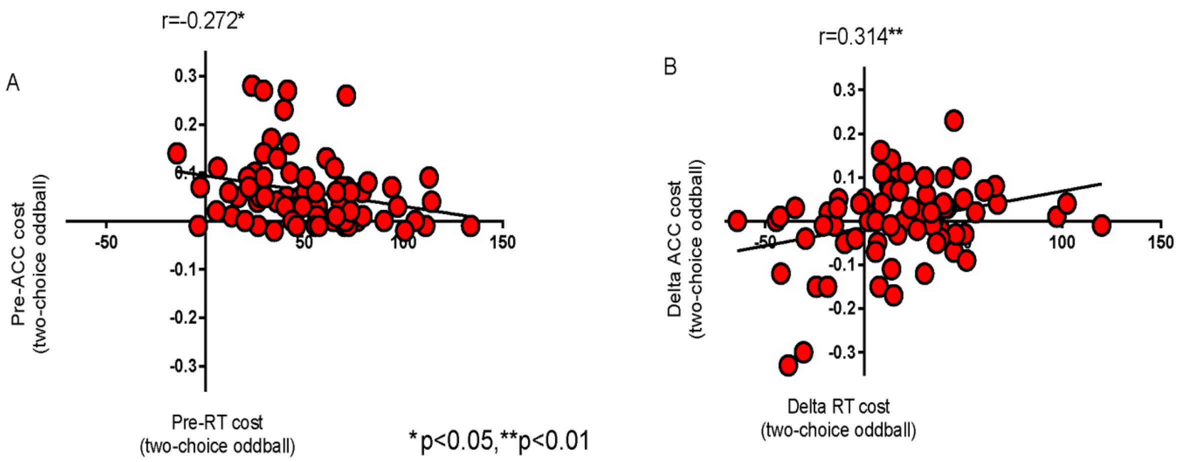

Supplemental material1: the scatterplots for the correlation between ACC cost (standard-deviant) and RT delay(deviant-standard) in the pretest, and the correlation between delta ACC cost (pre-post) and Delta RT delay (post-pre).

eFigure 1. Scatterplots for the Correlation Between ACC Cost and RT Delay in the Pretest and the Correlation Between Delta ACC Cost and Delta RT Delay

© 2020 Yuan J et al. *JAMA Network Open.*

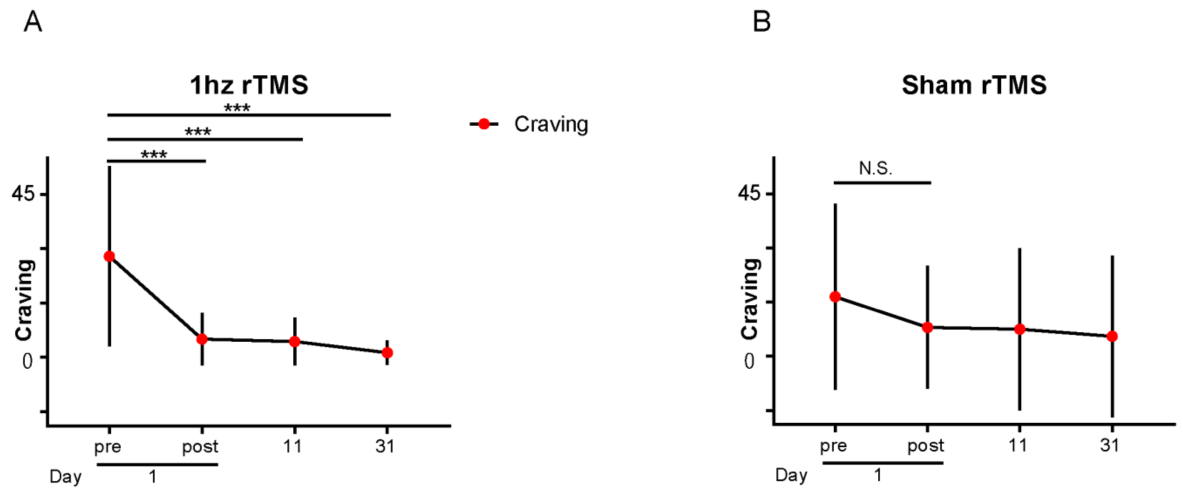

Supplemental material2: the schematic illustration (Mean±SD) of cue-induced craving varying as a function of the 1 Hz-rTMS protocol on Day 1, Day11 and Day 31 in real(left) and sham(right) group.

eFigure 2: Schematic Illustration of Cue-Induced Craving as a Function of the 1-Hz rTMS Protocol on Days 1, 11, and 31

***P < .001.

© 2020 Yuan J et al. *JAMA Network Open.*

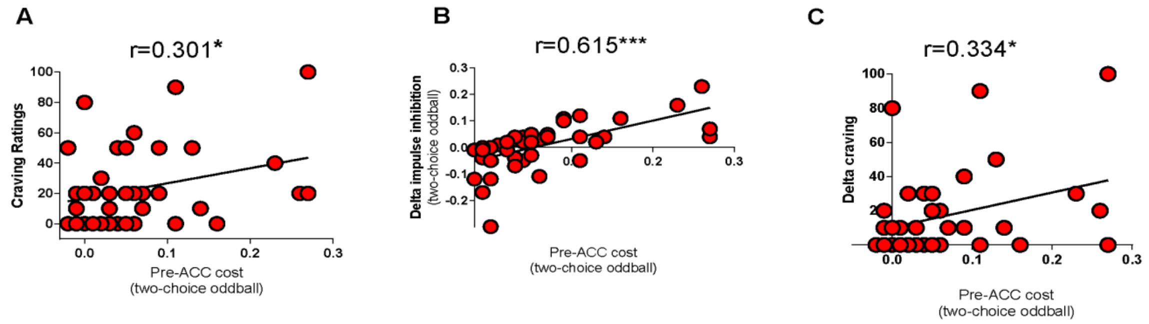

***p<0.05; ***p<0.001**

Supplemental material3: the scatterplots for the correlations between the ACC cost (standard-deviant) and cue-induced craving in the pretest (A), between pretest ACC cost and delta impulse inhibition(ACC cost$_{pre}$-ACC cost$_{post}$) （B), and between pretest ACC cost and delta craving (pre-post)(C).

eFigure 3. Scatterplots of the Correlations Between the ACC Cost and Cue-Induced Craving in the Pretest, Between Pretest ACC Cost and Delta Impulse Inhibition, and Between Pretest ACC Cost and Delta Craving

© 2020 Yuan J et al. *JAMA Network Open.*
