# Supplementary material for: Effect of Low-Frequency Repetitive Transcranial Magnetic Stimulation on Impulse Inhibition in Abstinent Patients With Methamphetamine Addiction: A Randomized Clinical Trial
Source: JAMA Netw Open. 2020 Mar 13;3(3):e200910. doi: 10.1001/jamanetworkopen.2020.0910 (PMC7070234; doi:10.1001/jamanetworkopen.2020.0910)
Supplement: Supplement 3. — Data Sharing Statement [file jamanetwopen-3-e200910-s003.pdf]

Data Sharing Statement

Yuan J, Liu W, Liang Q, Cao X, Lucas MV, Yuan T-F. Effect of low-frequency repetitive transcranial magnetic stimulation on impulse inhibition in abstinent patients with methamphetamine addiction: a randomized clinical trial. *JAMA Netw Open*. 2020;3(3):e200910. doi: 10.1001/jamanetwork.open.2020.0910

Data

Data available: Yes

Data types: Data dictionary

How to access data: Dr. Jiajin Yuan, Faculty of Psychology, Southwest University, E-mail:yuanjiajin168@126.com;

When available: With publication

Supporting Documents

Document types: None

Additional Information

Who can access the data: researchers whose proposed use of the data has been approved

Types of analyses: for meta-analysis, etc.

Mechanisms of data availability: with a signed data access agreement

Any additional restrictions: n/a
